# An *in vitro* analysis model for investigating the staining effect of various chlorhexidine-based mouthwashes

**DOI:** 10.4317/jced.53375

**Published:** 2017-03-01

**Authors:** Alain-Ayepa Kouadio, Xavier Struillou, Céline Bories, Jean-Michel Bouler, Zahi Badran, Assem Soueidan

**Affiliations:** 1 DDS, UFR d’odontologie. 1, Place A. Ricordeau, 44082, Nantes cedex2; 2 DDS, PhD, UFR d’odontologie. 1, Place A. Ricordeau, 44082, Nantes cedex2; 3 Prof, PhD, UFR d’odontologie. 1, Place A. Ricordeau, 44082, Nantes cedex2

## Abstract

**Background:**

There are different mouthwashes containing chlorhexidine in different concentrations, as well as various excipients. Chlorhexidine induce stains or discoloration in teeth and mucous membranes. The aim of this work was to design a model to reproduce *in vitro* staining associated with the use of different mouthwashes containing chlorhexidine.

**Material and Methods:**

We used as substrates of natural teeth and elephant ivory slices. Different incubation baths were conducted over 21 days in culture dishes at 37°C. At the beginning of experiment before incubation (D0) and after 21 days (D21) of incubation with different mouthwashes, pictures of substrates were taken in a standardized manner and an image analysis software was used to analyse and quantify the staining under the various conditions by using the 3 main colours (Red, Green, Blue, RGB).

**Results:**

The results of this work demonstrate a very good reproducibility of the protocol, and secondly, a different expression statistically significant of the primary blue colour. We suggest that for a given concentration of chlorhexidine, the staining effects may vary depending on the excipients used.

**Conclusions:**

This replicable model, easy to implement over a relatively short duration, can be used for evaluation of existing mouthwashes, and to test the excipients anti discoloration proposed by manufacturers.

** Key words:**In vitro, chlorhexidine, mouthwashes, dental stain, tooth discoloration.

## Introduction

Dental plaque is the main etiological factor for the two main dental diseases which are carious diseases and periodontal diseases. Numerous studies have shown that the rigorous control of dental plaque considerably minimizes progression of these diseases. Mechanical and chemical plaque control help maintain oral and dental health (1). During the periodontal treatment phase, practitioners may recommend the use of antimicrobial agents to inhibit plaque formation, treat gingivitis and periodontitis. These anti-microbial agents include metal salts, essential oils, phenols, fluorides, quaternary ammonium and biguanides. However, the list is not exhaustive.

Chlorhexidine digluconate, from the biguanide family, is considered to be the gold standard in plaque control therapies (2).

Chlorhexidine has very low toxicity and very high affinity for salivary proteins (3). Hydroxyapatite (the main component of enamel) is unable to retain chlorhexidine; however, enamel hydroxyapatite is permanently coated with a protective biofilm essentially made up of salivary glycoproteins. This suggests that the salivary and bacterial biofilm, present on the dental surface very early, plays an important role in chlorhexidine retention at the tooth’s surface. Hjeljord *et al.* (4) showed that chlorhexidine adsorption was significantly greater for proteins of high molecular weight of over 50 KDa. However, adsorption is almost non-existent for proteins of molecular weight under 20 KDa. These authors put forward the hypothesis that the adsorbed chlorhexidine is retained on the surfaces of the oral tissue (mucosa and dental) by electrostatic bonds. The bound molecules are then believed to be gradually replaced by calcium ions. The ability of chlorhexidine to bind to such an extent with proteins is one of the reasons which may explain its remanence despite circulation of saliva. According to Addy *et al.* (5), the reduction in micro-organisms in the saliva is believed to reach 90% for several hours after using a chlorhexidine-based mouthwash. Mendieta *et al.* (6) showed an anti-gingivitis effect after rinsing with chlorhexidine-based solutions twice a day for one week, and that the effects on plaque inhibition by chlorhexidine were significantly greater compared to the control group. This long-lasting antibacterial effect makes this molecule an excellent agent in the prevention and treatment of oral diseases of bacterial origin.

However, using chlorhexidine-based chemical agents causes numerous adverse effects, especially brownish coloration or staining at the surface of the teeth and gums after several days of use (7).

The growing aesthetic demand from patients is bringing industrialists to research and develop other types of mouthwash without adverse effects such as dental and mucosal staining. Examples of such include the addition of Anti-Discoloration System (ADS) to the mouth wash. Currently, there is no standardized protocol to test *in vitro*, the discoloration or staining effect of different mouthwashes

The purpose of this work is to test an *in vitro* analysis model for investigating the discoloration/staining effect of various chlorhexidine-based mouthwashes on enamel and ivory.

## Material and Methods

This is a comparative, non-randomised *in vitro* study which was conducted in several phases: creation of samples from human teeth and elephant ivory, development of incubation solutions, incubation in plastic containers, acquisition of images of the samples before incubation (D0) and after three weeks’ incubation (D21), and finally the quantification of substrate coloration by image analysis.

-Sample preparation 

Human teeth were extracted for various reasons at the dental care centre in Nantes (incisors, canines, premolars and molars). The teeth collected were treated with a sodium hypochlorite-based solution at 7°, then rinsed in distilled water and kept in a container filled with distilled water. The teeth were cut in two lengthwise using a BUEHLER IsoMet on speed setting 4 (out of 10). The largest diamond blade was used while irrigating with BUEHLER Cool 2 coolant.

**Figure 1:**
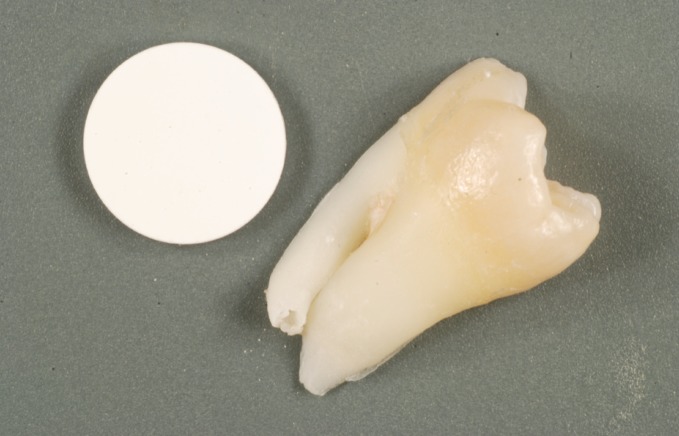
Lengthwise cross-section of a tooth next to a calcium phosphate pellet used to calibrate the photographs.

**Figure 2:**
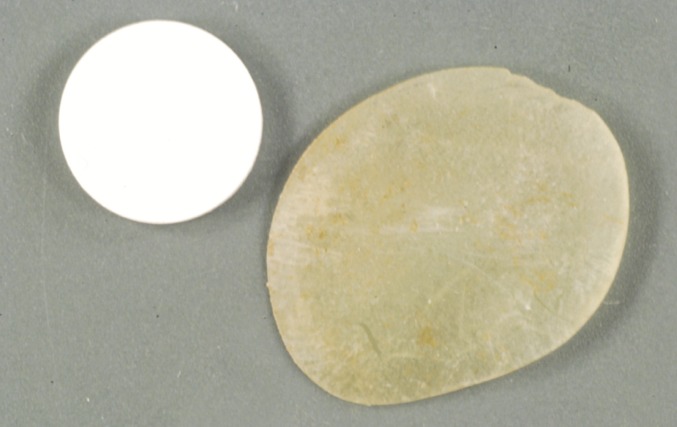
Ivory cross-section 1 cm in diameter and 300 µm thick next to a calcium phosphate pellet used to calibrate the photographs.

The teeth, quickly cleaned, dried and gently surface-wiped with compresses were adjusted and stuck to a plate with cyanoacrylate glue in order to be cut. A lengthwise tooth cut provides two enamel substrate samples (Fig. 1). African elephant ivory statues were collected from the Nantes customs agency and cut with a BUEHLER IsoMet on speed setting 4; the aim was to produce a piece measuring around 10 cm long and 1cm in diameter to make sample cutting easier. Each piece was then cut with another IsoMet, a LEICA SP1600, on speed setting 15 while irrigating with water. We produced pieces of ivory 1 cm in diameter and 300 microns in thickness (Fig. 2).

The sample surface was even given that they came from the same piece of ivory cut in the same conditions, which ensured sample reproducibility. However, the enamel of the teeth collected after extraction for various reasons naturally differed in terms of colour and surface condition. It should be emphasised nevertheless that samples were randomly distributed, which constitutes de facto randomisation, which should ensure fairly equivalent distribution for each condition.

Enamel thickness was not measured but represented half the crown. It should also be noted that staining was only looked for on the outer layer of the ivory and the enamel. We tested several ivory thicknesses (100, 200, 300, 500, and 600 microns) before selecting slices 300 microns thick. During this stage we were able to verify the quality of the slices produced under irrigation which, due to the low thickness (300 microns), were used to produce malleable pieces less rigid than thick pieces more likely to crack and break. It is also from this thickness that the alcohol contained in the mouthwashes no longer had a deforming effect on the pieces of ivory.

Three commercial chlorhexidine-based mouthwashes were tested.

* Solution A: an original mouthwash containing chlorhexidine (0.10%) / chlorobutanol (0.5%)

* Solution B: a mouthwash containing chlorhexidine (0.12%) associated to an Anti Discoloration System ®

* Solution C: a generic mouthwash containing chlorhexidine (0.10%) / chlorobutanol (0.5%) 

 * Solution D: Tea (positive control)

The tea was prepared according to the manufacturer’s recommendations. One tea spoon of dried tea was added per 100ml of water. The water was boiled to 95°C then infused for 4 minutes and left to cool at ambient temperature.

For each condition, 4 ml of mouthwash solution was placed in each well (box of 12 wells, cell culture plate Corning Incorporated®) using a graduated pipette. Then, for the series of mouthwash incubation solutions combined with the tea solution, 2 ml of tea solution was placed in each well in addition to the 4 ml mouthwash solution (final volume 6 ml).

We thus obtained 7 incubation solutions in total:

1) Solution A, pure alone (4 ml/well), 2) Solution A, pure combined with the tea solution (6 ml/well), 3) Solution B, pure alone (4 ml/well), 4) Solution B, pure combined with the tea solution (6 ml/well), 5) Solution C, pure alone (4 ml/well), 6) Solution C, pure alone combined with the tea solution (6 ml/well), 7) Solution D Tea solution (positive control) (4ml/well).

6 pieces of enamel and 6 pieces of elephant ivory were used for each type of incubation solution (n=6). There were 6 substrates for each condition to reduce the risk of having a high standard deviation in the event we had used a triplicate, which is the usual procedure for this type of calculation.

In order to remain efficient and organised, boxes of 12 wells were used for each mouthwash as follows: 6 wells for the mouthwash alone and 6 other wells for the mouthwash and tea solution.

We had 84 samples in total: six samples per substrate (2x6=12 enamel or elephant ivory) and 7 incubation solutions (7x12).

- Equipment for sample image acquisition before incubation on D0 followed by incubation.

o Twelve-well culture dishes, pliers, cup, graduated syringe, adhesive tape.

o Novasial® artificial saliva in 5 ml carton 

o Plastic stretch wrap

o Dry oven at 37°C 

o Camera (Nikon D80 and Sigma 1200 macro lens)

Camera stand set up in a dark room with grey background. The stand comprises two 12V lights on the left and two 12V lights on the right. Each light has 25W power. Four lights directed on a single sample attenuate shadow as far as possible as each of the lights attenuates the shadow thrown by the opposite light.

The camera was set at a constant distance for optimal magnification corresponding to the focal length of the macroscopic lens used on the light stand. The following settings were applied: manual mode, aperture 22 / 2.0k, iso sensitivity 200 and tungsten white balance. The images were saved in TIFF format. After careful cleaning of the substrate pieces using distilled water, each piece was dried in ambient air and placed on a grey background next to a white calcium phosphate pellet used to calibrate the acquisition parameters (grey background).

The grey background chosen for taking the photos prevents any interference with final photo quality. The samples were placed on a grey, matt background under special lighting to attenuate shade and shine from the enamel substrate further (due to the concavity of the crown and to its surface condition).

Images of the samples were acquired by the same operator (TG). 84 photos in total were taken.

Before incubation, each sample was then immersed in the artificial saliva for 1 minute to mimic the salivary biofilm. The samples were then incubated in the relevant wells. The containers were tightly closed with their lids and wrapped in plastic stretch wrap to prevent evaporation. They were then placed in an oven at 37°C for 3 weeks.

After three weeks’ incubation (D21), all samples were removed from the wells using pliers. Each sample was dried in ambient air to reduce shine, and then placed on a grey background in the same initial pre-incubation conditions for photos to be taken.

The photos were taken in exactly the same initial conditions (D0) and by the same operator (TG). 84 photos were taken on D21.

-Staining assessment (data collection and processing)

The samples were observed with the naked eye to look at the staining in the various test conditions.

Coloration was then assessed on the colour photos taken on D0 and D21 (168 colour photos in total). The Leica qwin 3 quantimet software was used to obtain the numerical value of each pixel in the 3 layers [red, green, blue (RGB)] making up colour. The assessment consisted of determining average grey scale (sum of grey scale values divided by the number of pixels considered to make up the area of interest). The advantage of having used grey scale was to be able to work per R B V colour layer separately from the image as a whole.

The analysis was performed in the following manner: Manual selection of the area to be analysed, selected using the cursor. The purpose of this selection is to have the most representative and largest area possible on each photo to be analysed. The assessment was then performed using the Leica qwin 3 quantimet software which gives the quantitative value for each colour [red, green, blue (RGB)]. The quantitative numerical data was collected in an Excel sheet for each substrate according to each condition.

This was repeated for the images taken on D0 representing the baseline or reference value, after three weeks’ incubation (D21).The difference in the D0-D21 numerical values was calculated for each colour, for each sample and for each substrate. The quantitative data will be used for the data analysis and for the statistical tests.

-Statistical analysis

Analysis of variance (ANOVA) and Fisher’s PLSD test were performed in order to compare the grey scale means observed on the various substrates (enamel and ivory). The differences were considered to be significant for *p*<0.05.

Distribution for all samples was normal, and no significant difference in variance was observed between the various groups (ANOVA).

## Results

During this research, the analysis method enabled us to collect quantitative information on the 3 main colours (RGB) for each sample. After overall and detailed analysis of the results for the two substrates (enamel and ivory) and to simplify matters, we opted for the blue colour which was the most discriminant of the 3 colors. The GB colours results will not be discussed in this work.

**Figure 3:**
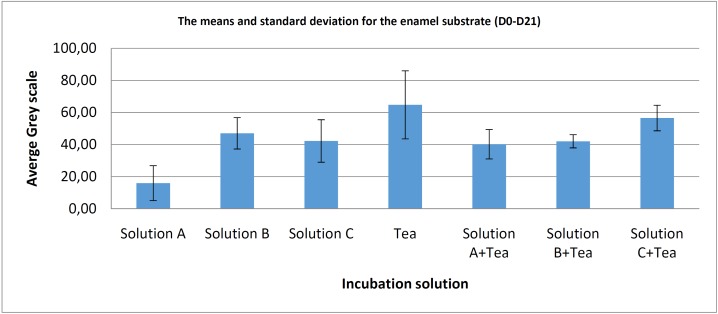
Bar chart showing the means and standard deviations for the differences in values (D0-D21) according to each incubation condition for the enamel substrate.

For the enamel ( Table 1, Fig. 3), calculation of the resulting means (n=6) represented by the standard deviation bars shows low standard deviation for all test conditions, which shows the good reproducibility of the method.

**Table 1:**
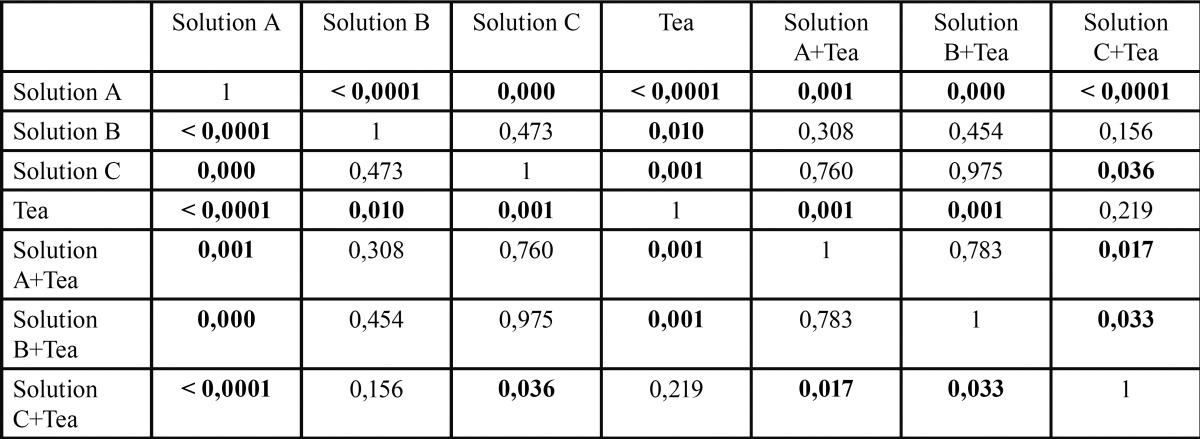
Statistical test summary (*P* value) for the enamel substrate and various incubation solutions.

It is important to specify that the quantitative results produced from the difference in the values between D21 and D0 are proportional to the colouring properties of each solution.

Comparison of the means for the 3 pure mouthwashes shows differences in the lowest values (D21- D0) and significant differences for solution A when compared to solution B (*p*<0.0001), and solution C (*p*<0.0001), which can be interpreted as being related to a lesser coloring effect for solution A among the 3 pure mouthwashes on this substrate. Addition of the tea solution leads to a significant difference in the case of solution A compared to solution A + tea (*p*=0.001), and solution C compared to solution C + tea (*p*=0.036), which logically reflects accentuation of the coloration in the presence of the tea solution, but this is not the case for solution B compared to solution B + tea which contains the ADS (*p*=0.454).

**Figure 4:**
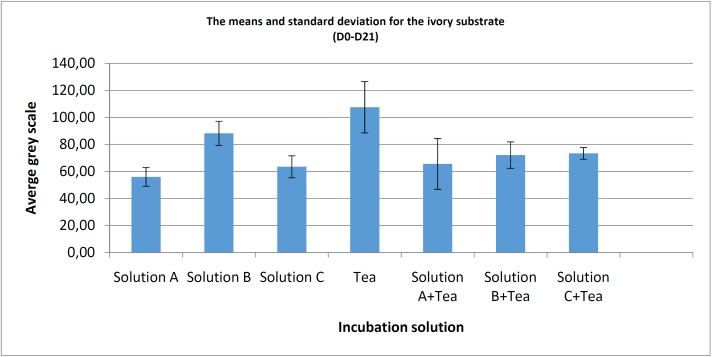
Bar chart showing the means and standard deviations for the differences in values (D0-D21) according to each incubation condition for the ivory substrate.

For the ivory ( Table 2, Fig. 4), calculation of the resulting means (n=6) also shows low standard deviation for all test conditions. Comparison of the means for the 3 pure mouthwashes shows differences in the lowest values with a statistically significant difference in favour of solution A, when it is compared to solution B (*p*<0.0001) however, there is no statistically significant difference with solution C (*p*=0.276). Addition of the tea solution shows lower values and a statistically significant difference in favour of solution B compared to solution B+ tea (*p*=0.023), but no significant difference when we compare solution A/solution A + tea (*p*= 0.165), nor for solution C when it is compared to the solution C + tea (*p*=0.155).

**Table 2:**
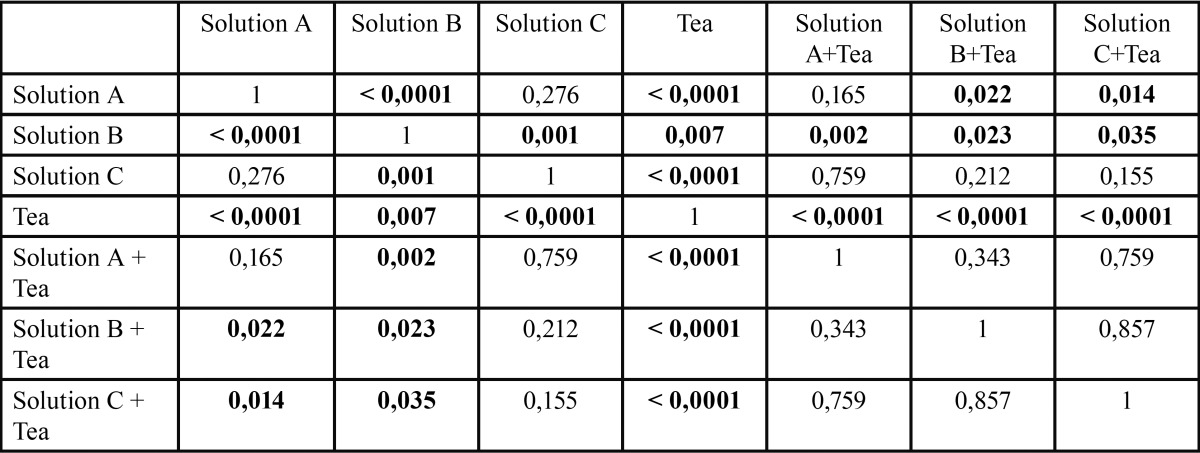
Statistical test summary (*P* value) for the ivory substrate and various incubation solutions.

Finally, it should be noted on the whole, that the results discussed in this research corroborates the visual observations (albeit subjective and non-quantitative) of the samples after drying.

## Discussion

First of all, it should be specified that the substrates (enamel and ivory) and incubation at 37°C, along with pre-incubation with an artificial saliva biofilm in this model, were chosen as we wished to recreate conditions representing clinical practice as far as possible.

In this study we opted for image capture using the Leica QWin3 software which makes it possible to work by RGB colour layer separately from the image as a whole. To that is added the fact that the substrates studied are too thick to be able to work in transmission (UV-visible spectrophotometer).

Consequently, although the 3 mouthwashes used do not have the same chlorhexidine content, it should be emphasised that according to the indications of each manufacturer, the usage concentration for each product leads to a different usage concentration therefore, the clinical usage concentration which served as reference in this study, is according to the recommendations of each manufacturer. Also, the excipients in the composition of each mouthwash are more or less different, which may also have an effect on the colouring properties of the chlorhexidine due to the chemical interactions with the various excipients.

Numerous studies have been conducted on this theme *in vitro* by Addy’s team (5,8-10). Perspex blocks have been widely tested in these *in vitro* studies. They are rectangular methyl polymethacrylate resin blocks measuring 30mm x10mm x3mm specially prepared for use in the spectrophotometer UV chamber. The results collected show a significant increase in coloration related to the use of chlorhexidine-based mouthwashes. However, few studies other than that by Addy’s team have been conducted.

The protocol set up as part of this research aimed at validating a test model using a different analysis technique on dental substrates, comes as close as possible to human clinical conditions of the use of mouthwashes.

Fine analysis of the quantitative results show low standard deviations for the same test condition, which leads us to suggest that this model is reproducible and sufficiently sensitive to be used as a study model for induced coloration and for the anti-discolourant properties of certain molecules. Also, the results differ according to substrate, which confers the model good sensitivity. The result of this research based on one of the 3 main colours (blue), show significant differences between the mouthwashes. We saw that solution A alone produces the lowest values (weakest coloring properties) on the enamel compared to the solution C and to solution B in a statistically significant manner. On the ivory, even if the solution A values remain the lowest, the difference is still statistically significant compared to solution B, but it is no longer significant compared to the solution C.

In the light of these observations, we can suggest that coloration varies according to the substrate tested, regardless of the mouth-wash used, which may be explained by the chemical nature of the substrate but also by the nature of the surface condition of each substrate which is different.

Quantitative differences in expression of the blue colour between the various mouthwashes could be related to the specific chemical composition of each mouthwash. This is especially obvious when we compare the solution A to solution C.

Three main mechanisms have been suggested to explain the origin of the chlorhexidine-induced coloration. Firstly, the non-enzymatic Maillard reaction, which is a series of fairly complex parallel and consecutive reactions that involves compounds with an amine function (peptides, amino acids, proteins) and reducing sugars (pentoses, hexoses), (11). Secondly, reactions with metal pigments in which, studies have demonstrated the ability of chlorhexidine to denature the proteins in the salivary biofilm (4). The proteins in the film are denatured by the formation of sulfhydryl groups after breaking of the disulphide bonds (11). There may therefore be an interaction with food-derived metal ions. The increase in the number of sulfhydryl groups leads to yellowing and metal sulphide groups cause brown coloration (12). Finally we can cite reactions with food products. Prayitno e*et al.* (8) and Addy *et al.* (13), demonstrated in vivo the colouring ability of chlorhexidine when it is combined with tea, red wine or coffee. This can be due either to the formation of pigmented components by reaction with the chlorhexidine (13), or to the high tannin compound content in tea and red wine especially (14). In effect, tannins are highly denaturing and red wine also contains many ferric compounds. Joiner *et al.* 2006 (15), studied chlorhexidine and black tea absorption *in vitro*. They concluded that chlorhexidine potentiated the colouring or staining effects of tea. After rinsing with chlorhexidine, coloration increased, but rapidly returned to initial state, whereas with tea, the effects were enhanced and return to initial state took longer.

In our study, we effectively saw an increase in the expression of blue coloration when the mouthwash solution was combined with tea, except for the Curasept for which addition of ADS appears to effectively neutralise the colouring effect of the tea. The ADS is a combination of sodium metabisulphite and ascorbic acid. Metabisulphite is an inorganic element used for its disinfectant and anti-oxidant properties. Ascorbic acid is also an anti-oxidant which reduces bacterial proliferation. Studies conducted by Solis *et al.* (16), compared coloration induced after using two chlorhexidine-based mouthwashes *in vivo*, with or without ADS. For these authors, there is no significant difference between the products, as much in plaque control as in coloration. Another *in vivo* study by Arweiler *et al.* (17) compared a mouthwash with alcohol-free chlorhexidine at 0.2% with ADS to a mouthwash at the same concentration with 7% ethanol, and to a placebo solution containing only 14% alcohol. They concluded that in addition to reducing coloration, ADS reduced the antibacterial effect of chlorhexidine on dental plaque; the ADS is believed to interact with the chlorhexidine molecules and to inhibit adhesion of the positive charges of the digluconate molecules on the enamel (17). This contradicts the *in vivo* results by Bernardi *et al.* (18), who demonstrated similar dental plaque control between the two mouthwashes, with and without ADS, and a significant reduction in coloration when using the solution with ADS. According to this study, the synergy of the two molecules contained in the ADS is believed to interfere with the staining mechanisms. Furthermore, Addy *et al.* (5) published an *in vitro* study using methyl methacrylate resin blocks. It compared mouthwashes with ADS and chlorhexidine concentrations of 0.12% and 0.2%, and did not find a difference in coloration between the two solutions with ADS. It is quite possible in this study that the 2-minute immersion in the solutions is not sufficient to show any difference in coloration between the two solutions, which shows the limits of this model. The *in vivo* study by Cortellini *et al.* (19) showed a significant difference in dental coloration when chlorhexidine was combined with the ADS, as we have seen in this study.

Among the limits to this research, we can cite enamel quality, the teeth used being taken from elderly patients with highly varied history and the enamel surface was not very even. However, it is fairly easy to prepare the ivory and its composition is very similar to that of enamel as, it keeps well over time. It is easy to prepare, sensitive to coloration and is a choice substrate for studying *in vitro* coloration.
